# Bimetallic Pd_96_Fe_4_ nanodendrites embedded in graphitic carbon nanosheets as highly efficient anode electrocatalysts†

**DOI:** 10.1039/c9na00317g

**Published:** 2019-08-19

**Authors:** Srabanti Ghosh, Sandip Bysakh, Rajendra Nath Basu

**Affiliations:** Fuel Cell and Battery Division, CSIR – Central Glass and Ceramic Research Institute 196, Raja S. C. Mullick Road Kolkata-700032 India ghosh.srabanti@gmail.com rnbasu@cgcri.res.in; Materials Characterization Division, CSIR – Central Glass and Ceramic Research Institute 196, Raja S. C. Mullick Road Kolkata-700032 India

## Abstract

A facile route to anchor a nanoalloy catalyst on graphitic carbon nanosheets (GCNs) has been developed for preparing high-performance electrode materials for application in direct alcohol fuel cells (DAFCs). Uniformly dispersed bimetallic Pd–Fe nanoparticles (NPs) with tunable composition have been immobilized on GCNs derived from mesocarbon microbeads (MCMBs) by a one-pot radiolytic reduction method. The Pd–Fe/GCN hybrid shows promising electrocatalytic activity for the methanol, ethanol, ethylene glycol, tri-ethylene glycol and glycerol oxidation reactions in alkaline medium. The as-prepared flower-shape Pd_96_Fe_4_/GCN nanohybrids have high mass activity for the ethanol oxidation reaction (EOR), which is ∼36 times (11 A per mg Pd) higher than that of their monometallic counterparts. Moreover, the onset oxidation potential for the EOR on the Pd_96_Fe_4_/GCN nanohybrids negatively shifts *ca.* 780 mV compared to that on commercial Pd/C electrocatalysts, suggesting fast kinetics and superior electrocatalytic activity. Additionally, chronoamperometry measurements display good long-term cycling stability of the Pd_96_Fe_4_/GCN nanohybrids for the EOR and also demonstrate only ∼7% loss in forward current density after 1000 cycles. The superior catalytic activity and stability may have originated from the modified electronic structure of the Pd–Fe nanoalloys and excellent physicochemical properties of the graphitic nanosheets. The present synthetic route using GCNs as the supporting material will contribute to further design of multimetallic nanoarchitectures with controlled composition and desired functions for fuel cell applications.

## Abstract 2

Bimetallic Pd–Fe nanoalloys with tunable composition immobilized on graphitic carbon by one-pot radiolytic reduction show high electrocatalytic activity for alcohol oxidation.

## Graphical abstract

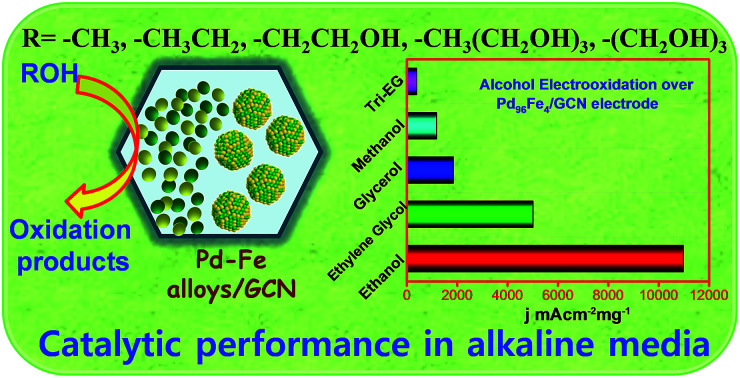

## 1. Introduction

Direct alcohol fuel cells (DAFCs) have drawn extensive attention for next generation energy conversion devices and have several advantages, including instantaneous recharging, lighter weight, high specific energy, environmental friendliness and a widespread range of applications, such as in portable electronic equipment, as residential clean energy power sources, and in fuel-cell-powered notebooks, computers, mobile phones, military equipment, *etc.*^1–3^ Catalysts are the key components for both the anodic oxidation reaction and the cathodic oxygen reduction reaction in fuel cells and up to now, Pt-based nanomaterials are considered as the most active electrocatalysts.^4–6^ However, the high cost, low abundance, poor reaction kinetics, and loss of electrochemical surface area of Pt catalysts has hindered the commercialization of fuel cells.^7^ As an alternative, researchers developed earth-abundant Pd metal-based electrocatalysts for alcohol oxidation in alkaline media.^8–10^ The engineering of the shape and composition of Pd-based materials at the nanoscale provides improved catalytic activity and stability.^8–12^ In this regard, bimetallic nanostructures display remarkably improved catalytic activity compared with the corresponding monometallic nanocrystals due to the modified electronic structures and ligand effects between the different components.^13,14^ Various bi/trimetallic Pd-based electrocatalysts (such as Pd–Ag, Pd–Pt, Pd–Ni, Pd–Cu, Pd–Au, Pd–Pt–Au, *etc.*) showed superior electrocatalytic activity for the methanol (MOR) or ethanol oxidation reaction (EOR).^15–21^ Very recently, our group demonstrated that conducting polymer nanofiber supported Pd based trimetallic nanoalloys are highly promising electrocatalysts for the EOR in alkaline media.^22^ Particularly, Pd or Pt-based core–shell-type noble metal nanostructures have been widely employed as efficient anode catalysts for the alcohol oxidation reaction.^23–31^ However, fabrication of such nanostructures involves complicated multistep processes, which make the process difficult to scale up. Moreover, these Pd-based anode electrocatalysts still suffer from low activity and high cost, which endorses the need for a facile strategy to produce advanced electrocatalysts. To obtain higher utilization efficiency of Pd in alcohol fuel electrooxidation, transition-metal-based catalysts, such as PdM (M = Sn, Cu, Ni, Co, *etc.*) alloy or core/shell NPs, were tested and they showed noticeable potential for enhancing the catalytic activity due to the optimized electronic and structural effects.^32–35^ So far, bimetallic Pt–Fe nanocrystals have been synthesized and they generally exhibit higher electrocatalytic activity. For example, Guo *et al.*^36^ developed a facile solution-phase self-assembly method to deposit FePt NPs on a graphene surface, and the resulting catalyst shows enhanced catalytic activity and durability for the ORR in acidic medium. Mohammad and co-workers reported a binary FeO_*x*_/Pt nanoanode for formic acid electro-oxidation and proposed that the catalyst's activation, conducted through a surface reconstruction for the Pt surface sites, makes the facets favourable for oxidation.^37^ In another example, PtFe nanodendrites with high-index facets showed higher reactivity for oxygen reduction.^38^ Very recently, Guo and co-workers^39^ fabricated ultrafine Pt–Fe nanowires for the oxidation of ethylene glycol and glycerol, and their activity was 3.9 and 2.5 times greater than that of commercial Pt/C, respectively. Although Pt–Fe has been considered as one of the best catalysts, its Pd-based counterpart (Pd–Fe) has not been well studied for the EOR in alkaline media. Notably, noble metal nanodendrites with a branched structure generally show better activity and durability for the catalytic reaction due to their large surface area, unusual interconnected as well as porous structure and fast mass transfer.^40–42^ To the best of our knowledge, there have been no reports on the preparation of bimetallic Pd–Fe nanodendrites and their electrocatalytic behaviour remains unknown so far.

Furthermore, the utilization of noble metal nanostructures can be improved by using carbonaceous porous materials as the most suitable catalyst supporting matrix due to their high electrical conductivity, chemical stability, large accessible surface area and rapid ion transfer rate.^43^ So far, various porous carbons with different morphologies have been widely investigated, such as common carbon blacks including acetylene black, Vulcan XC-72, ketjen black, porous carbon nanospheres, carbon nanosheets, carbon nanofibers, graphene, conducting polymers, *etc.*^44,45^ Among these carbon materials, two dimensional (2D) carbon nanomaterials such as graphene and 2D carbon nanosheets have great potential as electrode supports, having unique properties, including high electrical conductivity, large surface area and a porous layer structure.^46–48^ For example, Lee's group, Chen's group and Lu's group synthesized Pt–Pd alloy nanostructures supported on graphene, which exhibited high catalytic performance for the EOR.^49–51^ According to Chen's report, graphene enhances the catalytic activity of the Pt–Pd alloy nanostructures for the EOR due to the improved electrocatalyst–electrode electron transfer pathway. Xu's group found that graphene supported ternary PtAuRu alloys exhibited excellent electrocatalytic activity for ethanol oxidation.^52^ A series of multimetallic electrocatalysts have also been developed for alcohol oxidation based on a graphene support in order to adjust the chemical/electrical structures of the supported nanostructures, which can facilitate charge transfer and ionic interchange.^53–56^ Despite this success, graphene usually suffers from serious agglomeration and restacking because of strong van der Waals interlayer interactions, which certainly lower the specific surface area.^57,58^ Due to the weak interactions between surface graphene and metal species, it is difficult to get highly dispersed bimetallic NPs on graphene, and further modification of the graphene surface with electron-rich functional groups/sites may improve the binding ability of metal precursors. Hence, the synthesis and functionalization process of graphene are relatively complicated and high-cost, which limits its practical application.^59^ Interestingly, 2D carbon nanosheets with graphene-like layered structure materials possess high electrical conductivity, a large number of active sites and high effective surface area, which make them ideal as an electrocatalyst support.^60,61^ Many advanced techniques, including activation, helium ion bombardment, the template method, mechanical/chemical exfoliation, chemical vapour deposition, *etc.*, have been developed to fabricate carbonaceous nanosheets but establishing an efficient and facile synthesis methodology for 2D porous carbon nanosheets would be of great benefit for their large scale production.^62–65^ Mesocarbon microbeads (MCMBs) are one of the commercially available conducting carbon materials with good thermal and chemical stability and high crystallinity that are widely utilized for cost-effective electromagnetic interference shielding application, supercapacitors, and as an anode material in lithium-ion batteries and a supporting material for fuel cell applications.^66,67^ It is worth mentioning that there is no report in the literature on the synthesis of carbon nanosheets using MCMBs as the carbon source and this motivated us to develop a facile one-pot synthesis for metal deposited carbon nanosheets as multifunctional electrocatalysts.

Radiolysis is a promising alternative colloidal technique for the synthesis of nanoparticles with a narrow size distribution due to controlled particle nucleation and growth processes.^68,69^ Herein, we reported an efficient route to simultaneously fabricate Pd–Fe nanoalloys and deposit them on 2D graphene-like carbon nanosheets (GCNs) derived from MCMBs by a radiolytic method and reduction of Pd and Fe complexes. The highly dispersed bimetallic Pd–Fe NPs supported on graphitic nanosheets show exceptionally high catalytic activity toward alcohol electrooxidation in alkaline media, which has potential for application in fuel cells. The resulting metal alloy nanostructure and carbon nanohybrids were then characterized using common techniques, such as transmission electron microscopy (TEM), high-angle annular dark-field scanning transmission electron microscopy (HAADF-STEM), elemental mapping analysis, X-ray diffraction (XRD), and X-ray photoelectron spectroscopy (XPS) to confirm the formation of nanohybrids with controlled compositions by one-step radiolytic reduction.

## 2. Experimental section

### 2.1 Materials and reagents

Palladium(ii) acetylacetonate, iron(iii) acetylacetonate, 2-propanol, ethanol (≥99% for HPLC), KOH (97%), HCl, Nafion® suspension (5 wt%) and acetone were purchased from Sigma-Aldrich. Mesocarbon microbeads (MCMBs) were purchased from MTI Corporation, CA, USA. All of the reagents employed in the present study were used as received without further purification. Ultrapure water (Millipore System, 18.2 MΩ cm) was used as the solvent.

### 2.2. Synthetic procedures

#### Metal nanoparticle deposition on MCMBs

Metal nanoparticles were deposited on mesocarbon microbeads by using steady state gamma irradiation. In a typical experiment, 1 mg mL^−1^ MCMB solution was mixed with 1 mM palladium(ii) acetylacetonate, and iron(iii) acetylacetonate solution and then deaerated under a N_2_ flow. This final solution was irradiated in Co-60 gamma irradiation chamber at a dose rate of 10.8 kGy h^−1^ (dose, 55 kGy h^−1^). Finally, the mixture was centrifuged and washed with ethanol and the obtained precipitate was then dried at 60 °C. Similarly, bimetallic Pd–Fe/GCN with different compositions was prepared by selecting suitable mass ratios.

### 2.3. Characterization

High-resolution transmission electron microscopy (HRTEM) images were obtained with a Tecnai G2 30ST (FEI) high-resolution transmission electron microscope operating at 300 kV. The X-ray diffraction (XRD) patterns of the catalysts were recorded on a Philips X'Pert X-ray diffractometer (The Netherlands) with Cu Kα radiation (*λ* = 1.54056 Å). XPS measurement was performed on a PHI 5000 VersaProbe II spectrophotometer (Physical Electronics Inc., USA) with Al K_α_ (∼1486.6 eV) X-rays. Charge correction was made by taking C 1s spectra as the standard (284.5 eV). Raman spectra were collected using a JOBIN YVON HR800 Confocal Raman system employing a 632.8 nm laser beam. The spectra were recorded using a 20× objective and accumulated for 30 s. The composition of the nanohybrids was determined using a Spectro Ciros Vision inductively coupled plasma atomic-emission spectroscopy (ICP-AES) instrument, Spectro GmbH, Germany. The actual metal loading in each nanohybrid was determined by ICP-AES to be Pd_100_/GCN (9 ± 1%), Pd_96_Fe_4_/GCN (4 ± 0.15%), Pd_91_Fe_9_/GCN (3.4 ± 28%), Pd_85_Fe_15_/GCN (1.9 ± 0.5%), and Pd_77_Fe_23_/GCN (1.45 ± 0.12%).

### 2.4. Electrocatalytic experiments

All electrochemical measurements were carried out on a galvanostat–potentiostat (PGSTAT302N, Autolab, Netherlands). A conventional three-electrode system was used, with platinum wire as the counter electrode, a modified glassy carbon electrode (Carbone Lorraine 11 mm diameter) as the working electrode, and a Ag/AgCl (Hg/HgO) electrode as the reference electrode. The electrolyte solution was purged with argon gas in order to remove dissolved oxygen. Prior to surface coating, the surface of a glassy carbon electrode was polished with α-alumina powder. Then 5 μL of nanohybrid or commercial catalysts (20% Pd or Pt on Vulcan XC-72, JM) were deposited on the surface of the GC electrode and dried before electrochemical measurement. Cyclic voltammetry (CV) was carried out in 0.5 M KOH electrolyte and in the presence of alcohols such as ethanol, methanol, ethylene glycol, tri-ethylene glycol, and glycerol, at a scan rate of 50 mV s^−1^. Electrochemical impedance spectroscopy (EIS) measurements were recorded at a potential of 0.1 V. The charge transfer resistance (*R*_ct_) was measured by circle fitting from EIS measurement by applying an AC voltage with 10 mV amplitude in a frequency range from 0.1 to 10 kHz. The electrochemically active surface area (ECSA, m^2^ g^−1^ Pd) is usually determined using the formula ECSA = *Q*/(0.405 × *m*_pd_), where *Q* represents the coulombic charge corresponding to the reduction peak area of PdO (mC), and *m*_pd_ is the mass of Pd loading (mg) on the electrode, respectively. The charge required for the reduction of the PdO (*Q*_PdO-red_) monolayer has been considered as 405 μC cm^−2^.

## 3. Results and discussion

**Scheme 1:**
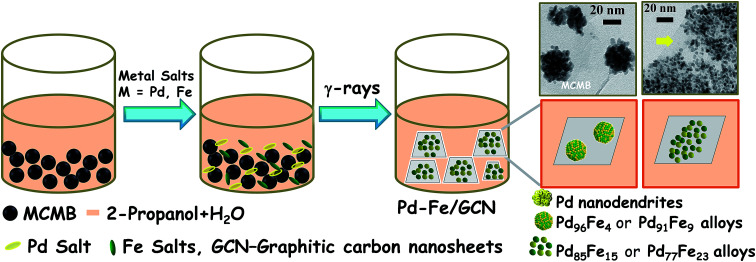
Schematic illustration of radiochemical synthesis of the Pd–Fe bimetallic alloy on carbon nanosheets.

Pd–Fe NPs were loaded on the graphitic nanosheets derived from mesocarbon microbeads by using a facile radiochemical method. Briefly, MCMBs were dispersed in a water–propanol mixture by ultra-sonication and mixed with palladium and iron salts in a pre-calculated Pd/Fe molar ratio at room temperature and irradiated at a dose rate of 10.8 KGy h^−1^ (Scheme 1). Notably, metal ions can easily be adsorbed on the interfaces of MCMBs and then be reduced to form NPs and MCMBs, subsequently being converted into graphitic carbon nanosheets (GCNs). Finally, graphitic carbon nanosheet supported bimetallic Pd–Fe NPs, denoted as Pd_1−*x*_Fe_*x*_/GCN (*x*, molar ratio of Fe), formed in the solution. The γ-irradiation creates electrons, H atoms, and hydroxyl radicals from the water, and Pd^2+^ and Fe^3+^ were reduced to form Pd–Fe nanoalloys.^70^

The metal ions were reduced to lower oxidation states or zero valence state by strong reductants, e_aq_^−^ and H, produced by water radiolysis, and oxidizing radiolysis products, particularly OH, were removed by adding scavengers (2-propanol) to prevent the back-oxidation of the zero-valence metal to a cation (eqn (1) and (2)). Due to the fast electronic process in radiolysis, the electron reduced a sufficiently high concentration of both metal ions to generate nanoalloy clusters at a high dose rate.^71,72^ Hence, electrons (e_aq_^−^) can reduce Pd(ii) and Fe(iii) ions into lower-state Pd(i) and Fe(ii) ions and then, neutral palladium and iron atoms, followed by growth of nuclei and co-deposition of metal atoms to form bimetallic alloy clusters (eqn (3)–(10)):^73,74^

1 H_2_O → e_aq_^−^, H_3_O^+^, H˙, OH˙, H_2_O_2_

2 (CH_3_)_2_CHOH + OH˙ (or H˙) → (CH_3_)_2_C˙OH + H_2_O (or H_2_)

3 e_aq_^−^ + M^+^ → M^0^ (M = Pd, Fe)

4 (CH_3_)_2_C˙OH + M^+^ → (CH_3_)_2_CO + M^0^ + H^+^

5 Pd^II^ + e_aq_^−^ → Pd^I^

6 Pd^II^ + (CH_3_)_2_C˙OH → Pd^I^ + (CH_3_)_2_CO + H^+^

7 2Pd^I^ → Pd^0^ + Pd^II^

8 *n*Pd^0^ → (Pd)_*n*_

9 Fe^III^ + e_aq_^−^ → Fe^II^ (*E*^0^ = +0.77 V)

10 Fe^III^ + 3e_aq_^−^ → Fe^0^ (*E*^0^ = −0.04 V)

**Fig. 1:**
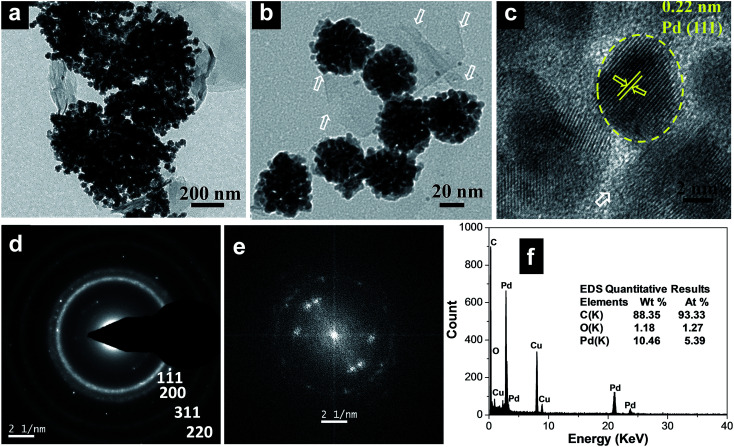
(a and b) Transmission electron micrographs of the Pd_100_/GCN nanohybrid at two different magnifications, (c) HRTEM image of palladium nanoparticles, (d) SAED pattern and (e) FFT and (f) EDS spectrum of the Pd_100_/GCN nanohybrid. The white arrows represent the thin sheets of graphitic carbon.

Clearly, the scanning electron microscopy (SEM) image of MCMBs exhibits porous 3D frameworks constructed from graphene layers with random open pores. The size of the MCMBs is in the range of 5–10 μm, while some smaller ones are less than 1 μm in size as shown in Fig. S1a, ESI.† After radiolysis, MCMBs converted into graphene-like carbon nanosheets as shown in transmission electron microscopy (TEM) and high resolution TEM (HRTEM) images (Fig. S1b and c, ESI†), which show ultrathin structures with a highly crumpled morphology. After radiochemical reduction of the Pd complex in the presence of MCMBs, the reaction leads to the formation of spherical Pd assembled nanostructures deposited on graphitic carbon nanosheets. The representative TEM images of the Pd_100_/GCN nanohybrid in Fig. 1a and b show that the Pd nanoflowers with an average particle-agglomerate size of ∼40 nm are uniformly deposited on the carbon nanosheet support. The HRTEM image (Fig. 1c) shows that each nanostructured agglomerate is composed of many single crystalline grains or nanocrystals. The interplanar distance in the lattice fringes of one such nanocrystal has been measured to be 0.22 nm, which corresponds to the (111) plane of metallic Pd. The selected area electron diffraction (SAED) pattern recorded from one of the Pd nanostructures shows diffraction rings corresponding to the various lattice planes of the face centred cubic crystal structure of palladium (Fig. 1d).^11,12^

Furthermore, the fast Fourier transform (FFT) as given in Fig. 1e, obtained from the HRTEM image (Fig. 1c), shows three pairs of bright spots corresponding to the (111) and (200) lattice fringes of cubic Pd in three different nanocrystals, suggesting that the flower-like individual nanoclusters are polycrystalline in nature and consist of fine Pd nanocrystals. The formation of such metal nanoflower structures by γ-radiation can be rationalized through the slow reduction kinetics particularly at the seeding stage which leads to the formation of 3D nanostructures.^75^ Energy-dispersive X-ray spectroscopy (EDS) analysis shows the presence of C and Pd (Fig. 1f).

**Fig. 2:**
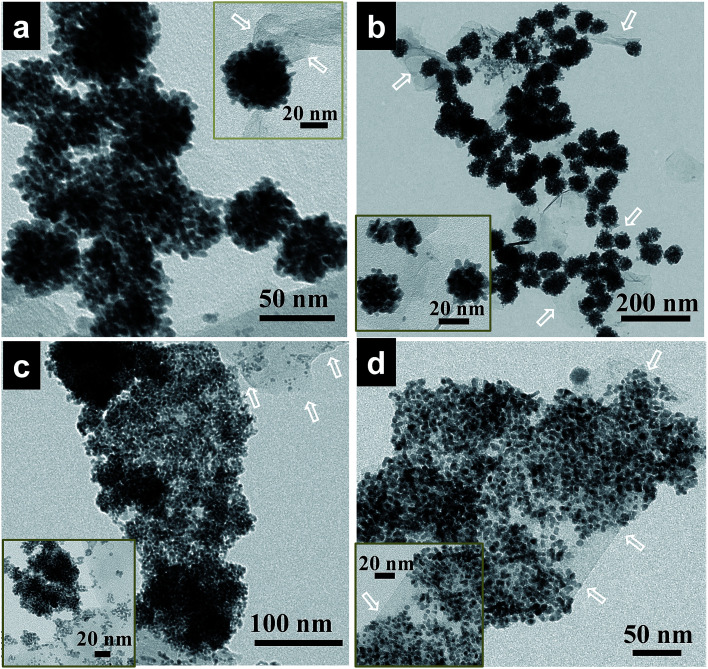
TEM images of Pd–Fe nanoalloys deposited on graphitic carbon nanosheets. (a) Pd_96_Fe_4_/GCN, (b) Pd_91_Fe_9_/GCN, (c) Pd_85_Fe_15_/GCN, and (d) Pd_77_Fe_23_/GCN nanohybrids. Inset: the corresponding Pd–Fe nanoalloys at high magnification. The white arrows represent the thin sheets of graphitic carbon.

Pd–Fe bimetallic nanoparticles with different compositions were also prepared using MCMBs as a supporting material through a reaction similar to that used for the Pd nanoflowers, where the amount of Pd and Fe precursors (Pd : Fe, 95 : 5, 90 : 10, 85 : 15, and 75 : 25) in the initial solution was varied while keeping the total volume of the solution unchanged. Interestingly, under similar synthetic conditions of the nanoalloy, only by enhancing the concentration of Fe precursors, which can be used as the shape controller, Pd–Fe alloy nanoflowers can be obtained with up to 9 at% Fe. As displayed in Fig. 2a and b, the as-prepared hybrids consist of Pd–Fe bimetallic nanoflowers with an average particle size of 50 nm and 54 nm, respectively.

**Fig. 3:**
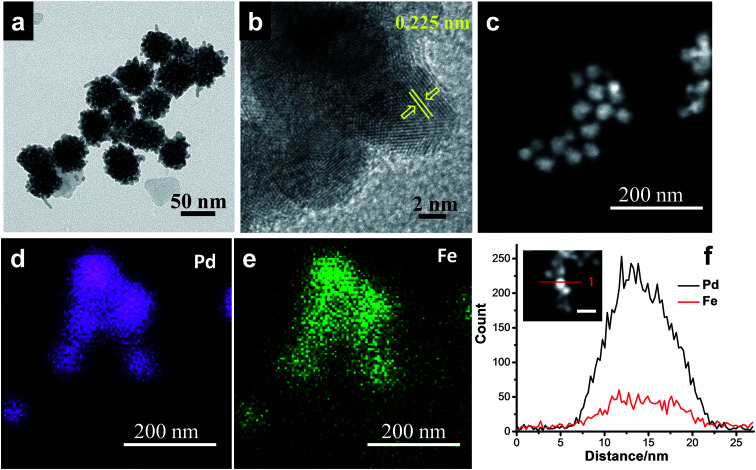
(a) TEM, (b) HRTEM, and (c) HAADF-STEM images, and (d and e) elemental mapping and (f) EDS line scanning profiles of the Pd_91_Fe_9_/GCN nanohybrids.

With further increase of Fe concentration from 10 to 25%, strikingly uniform sized spherical nanoclusters of Pd–Fe alloy (∼3.3 nm and ∼5 nm for Pd_85_Fe_15_ and Pd_77_Fe_23_ compositions, respectively) formed on the carbon nanostructures as shown in Fig. 2c and d. TEM and high angle annular dark-field scanning TEM (HAADF-STEM) images of the Pd_91_Fe_9_/GCN nanohybrids reveal the random distribution and severe aggregation of smaller NPs with the nanoflower structure (Fig. 3a).

The fringes in the HRTEM image (Fig. 3b) are separated by 0.225 nm, close to the (111) lattice spacing of the L1_0_ ordered structure of Pd–Fe. The superlattice structure resulting from L1_0_ type atomic ordering can be clearly seen in the HRTEM image of the Pd–Fe nanoparticles, which is consistent with earlier reports.^76,77^ It has been proposed in the literature that the L1_0_ superlattice crystal structure results from the alternate stacking of Fe and Pd in the (001) direction in the L1_0_ type ordering of the two different atomic species and the (220) atomic planes also possess an alternate stacking sequence of Fe and Pd in the (110) direction. In addition, the HAADF-STEM image (Fig. 3c) together with the EDS spectrum-image elemental mapping analysis of Pd (Fig. 3d) and Fe (Fig. 3e) shows that Fe (violet) is distributed on Pd atoms (green) throughout the whole area in the 3D alloy nanostructures. Hence, the STEM-EDS elemental mapping analysis within the resolution of the TEM used in the present work suggests that the elements Pd and Fe are distributed with fair uniformity within the nanoflowers. The bulk composition of the as-synthesized nanohybrids was determined by inductively coupled plasma atomic emission spectroscopy (ICP-AES) analysis and is in agreement with our initial proposed composition (Table S1†). Similarly, the EDS line scanning profiles across a single particle (Fig. 3f, inset) also reveal that the nanoflower consists of Pd and Fe.

Upon further increasing the precursor amount of Fe in the initial solution, spherical nanoparticles are formed instead of nanoflowers or nanoclusters. In contrast, the TEM and HAADF-STEM images of the Pd_77_Fe_23_/GCN nanohybrids indicate the uniform dispersion of Pd_77_Fe_23_ bimetallic alloy nanoparticles on the GCNs with an average particle size of 5 ± 1 nm (as shown in Fig. S2a and c, ESI†) and Fig. S2b† shows the typical HRTEM image of a single nanoparticle. The interval between the two lattice fringes is 0.225 nm, which corresponds well to the (001) plane of the ordered Pd–Fe L1_0_ structure and is consistent with the ideal atomic arrangement.^78^ The HAADF-STEM image and STEM-EDS elemental mapping suggest that the nanoparticles consist of Pd and Fe (Fig. S2c–e, ESI†). The results of both ICP-AES and EDS show that the nanoparticles are made of Pd and Fe. From ICP-AES analysis, the atomic ratio of Pd and Fe was almost 3.3 : 1, close to the theoretical proportion of 3 : 1. Moreover, the EDS line scanning profiles across a single particle (Fig. S2d, ESI†) also illustrates the formation of a uniform alloy composition within the individual Pd–Fe nanoparticles.

**Fig. 4:**
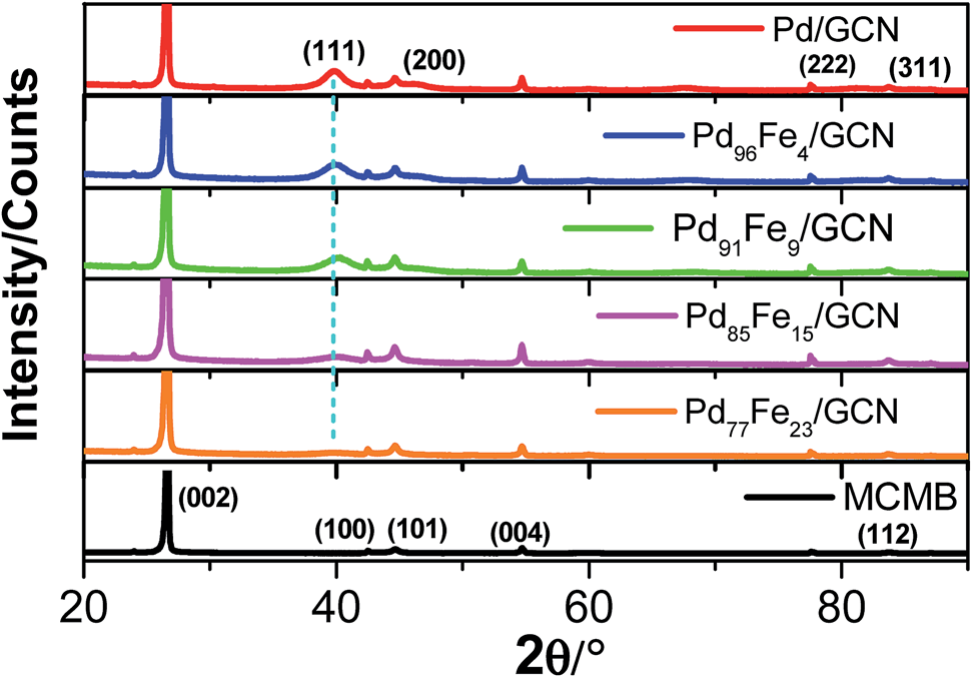
XRD patterns of the nanohybrid samples compared with that of MCMBs.

The X-ray diffraction (XRD) pattern of pristine MCMBs shows a very sharp diffraction peak at 2*θ* = 26° which is the characteristic peak of graphite and corresponds to the diffraction of the (002) plane, with the interlayer distance, *d*_002_ = 0.337 nm (Fig. 4).^79^

After radiolytic reduction, the strong diffraction pattern observed in all nanohybrids indicates the presence of graphitic carbon. The XRD patterns of the as-synthesized Pd–Fe/GCN shows the characteristic peaks of Fe–Pd particles that belong to a chemically disordered cubic structure. The (111) peak position in Fe–Pd appears at a slightly higher scattering angle compared to that of Pd nanoparticles, indicating the formation of a Pd–Fe alloy.^80,81^

**Fig. 5:**
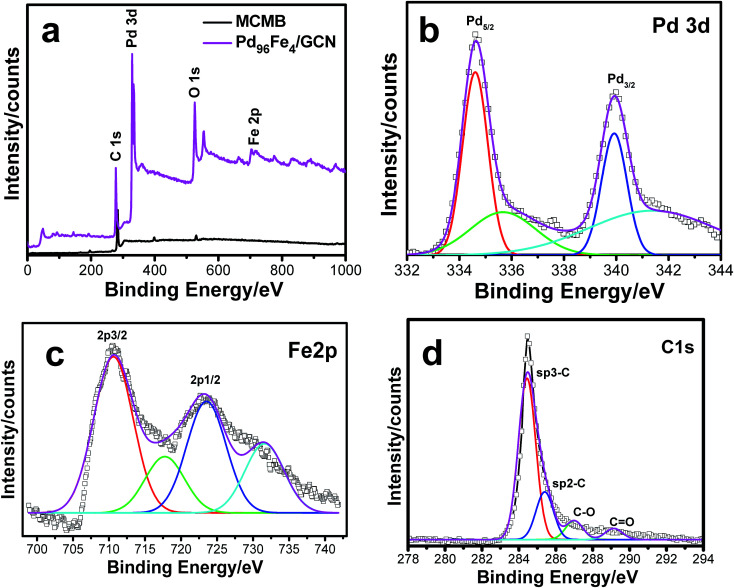
(a) XPS pattern for the MCMBs and as-prepared Pd_96_Fe_4_/GCN nanohybrids, and (b–d) magnified XPS spectra for (b) Pd 3d, (c) Fe 2p and (d) C 1s.

XPS analysis of the as-synthesized Pd–Fe/GCN was conducted to elucidate the composition and oxidation state of metal present in the nanohybrids as shown in Fig. 5. The wide scan XPS spectrum of the Pd_96_Fe_4_/GCN nanohybrids (Fig. 5a) reveals the presence of C, O, Pd, and Fe. The signals at 335.3 and 340.4 eV correspond to the Pd 3d_5/2_ and Pd 3d_3/2_ levels of Pd^0^, while the signals at small doublets around 336.7 and 342.1 are associated with the Pd 3d_3/2_ and Pd 3d_5/2_ levels of PdO (Fig. 5b). The Fe 2p spectrum is composed of Fe 2p_3/2_ and Fe 2p_1/2_ with binding energy at 710.6 eV and 723.9 which further confirmed the presence of di- and trivalent iron atoms as shown in Fig. 5c.

The satellite peaks of 719 eV and ∼732 eV at higher binding energy correspond to Fe^3+^ in α-Fe_2_O_3_ due to charge transfer as reported earlier.^82,83^ Moreover, the XPS peak at 285–286 eV corresponding to the C 1s region was attributed to carbon nanosheets (Fig. 5d). The Raman spectrum of the metal nanoparticle deposited graphitic nanosheets shows major peaks of D and G bands at 1346 nm and 1576 nm, respectively, which are normally present in graphitic materials as shown in Fig. S3, ESI.† Graphite basically consists of stacks of sp^2^ bonded planar graphene sheets. The D band arises from scattering of phonons which are associated with the structural defects and the G band is associated with the E_2g_ species of hexagonal graphite's infinite single crystal.^84,85^ Hence, Raman spectra imply the presence of graphitic structures within the nanohybrids.

**Fig. 6:**
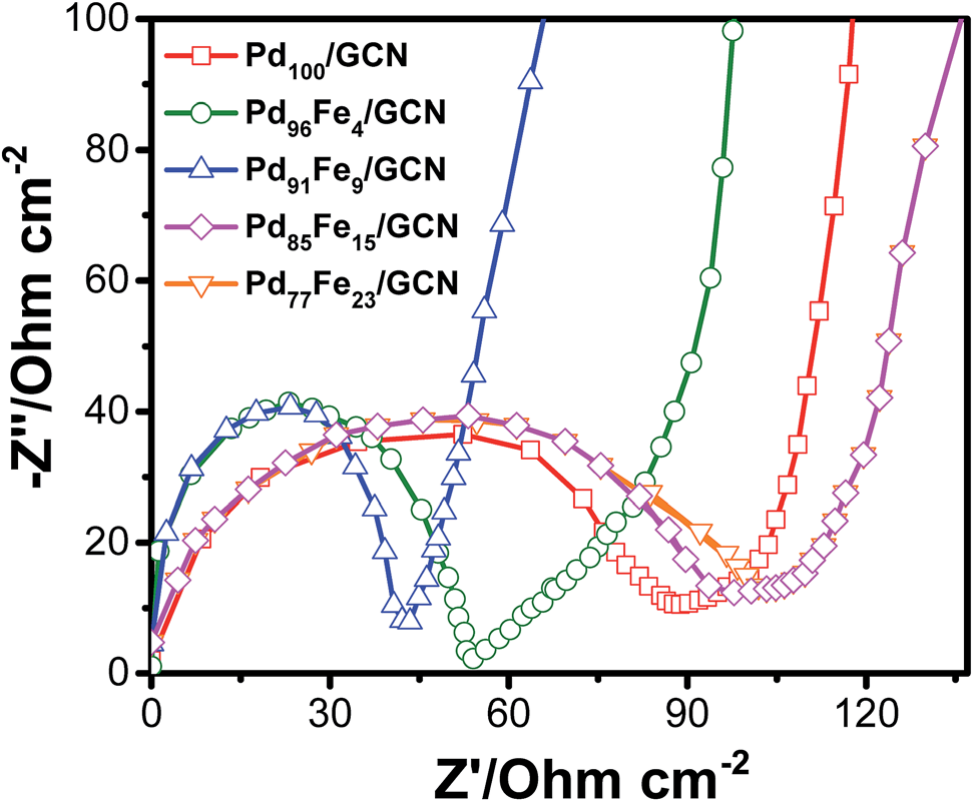
Nyquist plots of the Pd_100_/GCN, Pd_96_Fe_4_/GCN, Pd_91_Fe_9_/GCN, Pd_85_Fe_15_/GCN and Pd_77_Fe_23_/GC nanohybrids. Nyquist plot analysis indicates that the Pd_96_Fe_4_/GCN anode with small charge transfer resistance would be an outstanding anode material for DAFCs.

To examine the conductivity of the as-prepared anode materials, electrochemical impedance spectroscopy was employed to study the electrochemical behaviour and the charge-transfer resistance. Each Nyquist curve is composed of a well-defined semicircle followed by a straight line which represents the charge transfer resistance (*R*_ct_) and the solution resistance (*R*_s_) at the electrode/electrolyte interface, respectively, as shown in Fig. 6.^86,87^

The *R*_ct_ values corresponding to the diameter of the semicircles follows the order Pd_77_Fe_23_/GCN (∼103 ohms) > Pd_85_Fe_15_/GCN (∼94 ohms) > Pd_100_/GCN (∼84 ohms) > Pd_91_Fe_9_/GCN (∼54 ohms) > Pd_96_Fe_4_/GCN (∼41 ohms). The Pd_96_Fe_4_/GCN nanohybrid exhibited much lower charge-transfer resistance, which was half that of Pd_100_/GCN under similar electrolyte solution and experimental conditions. This suggests the effectiveness of bimetallic Pd–Fe NPs with a particular composition in enhancing the charge transfer ability of the Pd based nanohybrid. Hence, EIS results demonstrate that charge flow between the Pd_96_Fe_4_/GCN electrocatalyst and electrolyte has a faster electron transfer rate that can facilitate the electrochemical oxidation of alcohol compared to the Pd_100_/GCN.

**Fig. 7:**
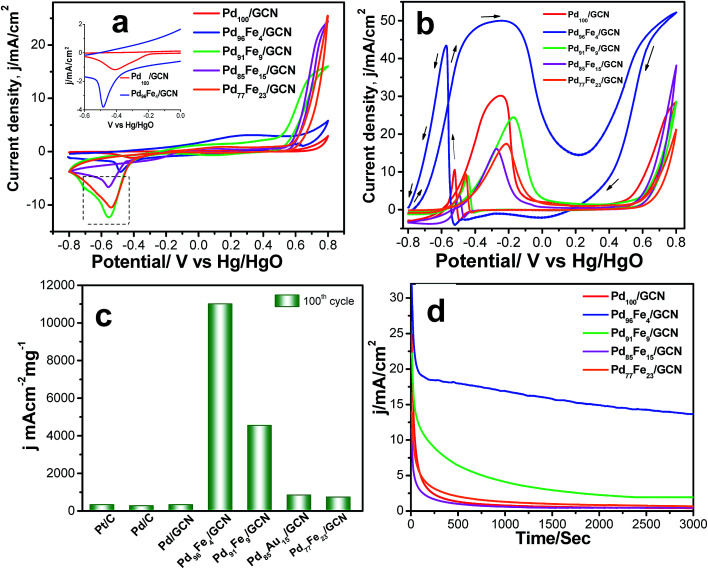
(a) CVs of the Pd_100_/GCN, Pd_96_Fe_4_/GCN, Pd_91_Fe_9_/GCN, Pd_85_Fe_15_/GCN, and Pd_77_Fe_23_/GCN nanohybrids in 0.5 M KOH at a scan rate of 50 mV s^−1^. (b) CVs for the electrocatalytic oxidation of 0.5 M ethanol at the 100^th^ cycle by Pd_100_/GCN (red solid line), Pd_96_Fe_4_/GCN (blue solid line), Pd_91_Fe_9_/GCN (green solid line), Pd_85_Fe_15_/GCN (pink solid line), and Pd_77_Fe_23_/GCN (orange solid line) nanohybrids at a scan rate of 50 mV s^−1^ in 0.5 M KOH. (c) Comparative values of mass activity of Pd/C, Pd/C, Pd_100_/GCN, Pd_96_Fe_4_/GCN, Pd_91_Fe_9_/GCN, Pd_85_Fe_15_/GCN, and Pd_77_Fe_23_/GCN electrodes for ethanol oxidation in alkaline medium. (d) Chronoamperometric curves for the ethanol oxidation at a constant potential of −0.22 V *vs.* Hg/HgO on Pd_100_/GCN, Pd_96_Fe_4_/GCN, Pd_91_Fe_9_/GCN, Pd_85_Fe_15_/GCN, and Pd_77_Fe_23_/GCN electrodes.

The cyclic voltammogram (CV) profiles for the Pd_100_/GCN, Pd_96_Fe_4_/GCN, Pd_91_Fe_9_/GCN, Pd_85_Fe_15_/GCN, and Pd_77_Fe_23_/GCN nanohybrid based electrodes clearly display peaks for hydrogen adsorption and desorption (H_ad_/H_des_), oxide formation, and oxide reduction.^10^ For Pd_100_/GCN, a peak at around −0.40 V in the first region of potential arises due to the adsorption of OH^−^ ions as evident from Fig. 7a.

Notably, the peak current in the region is higher for the alloys in comparison to the pure Pd indicating possible transmetalation reaction.^17^ The catalytic activity of four different compositions of Pd_1−*x*_Fe_*x*_/GCN (*x*, molar ratio of Fe) for the ethanol oxidation was evaluated in alkaline medium. The catalytic activity is highly dependent on the Pd/Fe molar ratio (Fig. 7b). The monometallic Fe_100_/GCN catalysts show no activity for ethanol oxidation (Fig. S4, ESI†), while the incorporation of Pd with Fe together results in a significant enhancement in catalytic activity. The ECSA of Pd_100_/GCN, Pd_96_Fe_4_/GCN, Pd_91_Fe_9_/GCN, Pd_85_Fe_15_/GCN, and Pd_77_Fe_23_/GCN has been calculated to be 15.6 m^2^ g^−1^, 80.5 m^2^ g^−1^, 28.5 m^2^ g^−1^, 23.5 m^2^ g^−1^, and 21 m^2^ g^−1^, respectively. The value of ECSA for Pd_96_Fe_4_/GCN has been found to be about 5.3 times higher than the value obtained for Pd_100_/GCN, which may be attributed to nanodendritic structures of metal NPs, and the synergistic effects between the metal NPs and graphitic surface.

Notably, Pd_96_Fe_4_/GCN shows exceptionally high catalytic activity toward the electrooxidation of ethanol with a high mass activity of 11 008.2 mA cm^−2^ mg^−1^, one of the highest values reported thus far (Fig. 7c and Table 1). In contrast, Pd_100_/GCN, Pd_91_Fe_9_/GCN, Pd_85_Fe_15_/GCN, and Pd_77_Fe_23_/GCN display reduced activity for ethanol oxidation under similar conditions (Fig. 7b and c). The catalytic performance of the catalysts follows the order Pd_96_Fe_4_/GCN (36-fold) > Pd_91_Fe_9_/GCN (15-fold) > Pd_85_Fe_15_/GCN (2.7-fold) > Pd_77_Fe_23_/GCN (2.3-fold) > Pd_100_/GCN (selected as the reference). Such remarkably high activity of Pd–Fe NPs supported on GCN is attributed to the synergistic effect between Pd and Fe atoms, and the strong interactions between the graphitic surface and metal species through graphene layers.

**Table 1:** Comparison of the electrochemical performance of Pd_100_/GCN, Pd_96_Fe_4_/GCN, Pd_91_Fe_9_/GCN, Pd_85_Fe_15_/GCN, and Pd_77_Fe_23_/GCN nanohybrids for the alcohol oxidation in alkaline medium

Fuel	Materials	*J*_f_ (mA cm^−2^)	*J* (mA cm^−2^ mg^−1^)	*J*_b_ (mA cm^−2^)	Ratio (*J*_f_/*J*_b_)	Onset potential (mV)
Ethanol	Pd_100_/GCN	30.2	305.6	10.7	2.8	657
	Pd_96_Fe_4_/GCN	49.8	11 008.2	43.6	1.14	780
	Pd_91_Fe_9_/GCN	24.5	4531	9.1	2.69	742
	Pd_85_Fe_15_/GCN	19.3	816.6	3.9	4.17	590
	Pd_77_Fe_23_/GCN	17.4	708.2	9.4	1.85	581
Methanol	Pd_96_Fe_4_/GCN	5.4	1193	1.01	5.32	320
Ethylene glycol	Pd_96_Fe_4_/GCN	22.6	5028	10.54	2.17	313
Tri-ethylene glycol	Pd_96_Fe_4_/GCN	1.29	403	1.35	0.95	384
Glycerol	Pd_96_Fe_4_/GCN	8.4	1860	1.47	5.69	241

To test the durability and recyclability of the nanohybrid, the Pd_96_Fe_4_/GCN anode was tested at −0.22 V in 0.5  M ethanol over 3000 s *via* the chronoamperometric method (Fig. 7d). Initially, all catalysts exhibited a pronounced current decay up to 250 s due to accumulation of poisonous intermediates; then the current density attained a steady state for Pd_96_Fe_4_/GCN indicating that the Pd_96_Fe_4_ NPs form a very stable film on the glassy carbon electrode surface and display stable electrocatalytic performance towards ethanol oxidation. However, for other electrodes, the current density significantly decayed in the first 200 s, and finally reached the zero value. The presence of graphitic carbon nanosheets as well as assembled Pd_96_Fe_4_ nanoalloy structures increases the stability of the anode catalysts for ethanol oxidation. No change in catalytic activity was observed over 1000 cycles (Fig. S5, ESI†). Remarkably, XRD, SEM and TEM analyses of Pd_96_Fe_4_/GCN after the catalytic reaction show no significant changes in size, distribution and structure suggesting that the Pd_96_Fe_4_/GCN catalyst possesses high stability under the current experimental conditions (Fig. S6b–d, ESI†). The superior stability was further confirmed by inductively coupled plasma atomic emission spectroscopic (ICP-AES) analysis, where no Pd and Fe were detected in the electrolyte following ethanol electrooxidation for 1000 cycles.

**Fig. 8:**
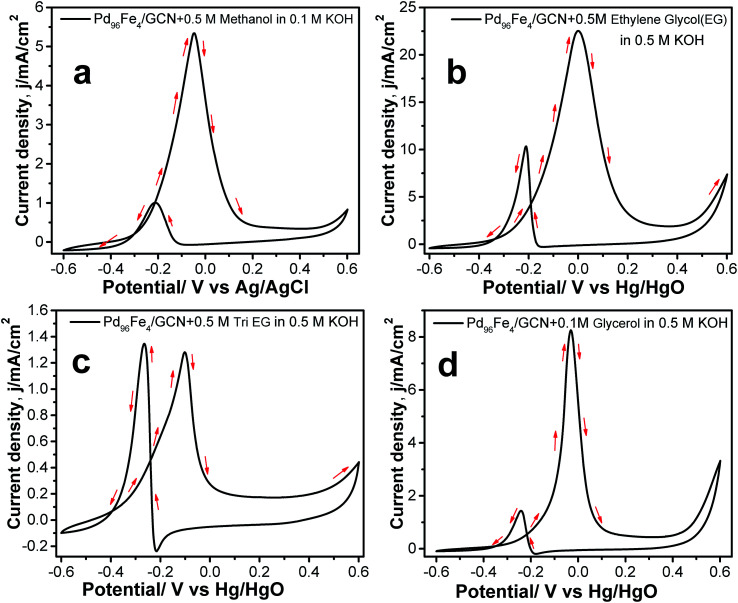
CVs for the electrocatalytic oxidation of (a) 0.5 M methanol in 0.1 M KOH, (b) 0.5 M ethylene glycol, (c) 0.5 M tri-ethylene glycol, and (d) 0.5 M glycerol at the 100^th^ cycle by Pd_96_Fe_4_/GCN in 0.5 M KOH at a scan rate of 50 mV s^−1^.

In order to explore the potential applications of the as-prepared catalysts, the methanol, ethylene glycol, tri-ethylene glycol, and glycerol oxidation reactions were also tested in alkaline solution. The CV profiles of all fuel oxidations display well-defined anodic peaks in the forward and reverse scans using Pd_96_Fe_4_/GCN as the anode catalyst (Fig. 8a–d). The highest mass activity was 11.008 A mg^−1^ metal (normalized by the mass of metal) obtained for ethanol oxidation, followed by that for ethylene glycol oxidation using the Pd_96_Fe_4_/GCN electrode (Table 1). This superior catalytic activity of Pd_96_Fe_4_/GCN toward the ethanol oxidation reaction suggests that optimizing the alloy composition can effectively enhance the overall electrocatalytic activity of the alloy. Notably, the obtained catalytic activity of the Pd_96_Fe_4_/GCN is even superior to that of other Pt or Pd group-based electrocatalysts for the ethanol oxidation reaction in alkaline media (Table S2†). The significantly negative shift of the onset potential and high anodic peak current for the EOR on the Pd_96_Fe_4_/GCN electrode suggest outstanding electrocatalytic activity for direct ethanol fuel cells. The Pd_96_Fe_4_/GCN generates a mass activity of 5.02 A mg^−1^ metal for ethylene glycol oxidation, which is much higher than that of PdPt nanowires,^88^ Pd nanodendrites supported on reduced graphene oxide,^89^ Pd-(Ni–Zn)/C,^90^ and Pd-decorated FeCo@Fe/C core–shell nanocatalysts,^91^ and is even comparable to that of trimetallic Pd–Au–Ag catalysts.^92^ A similar tendency is also observed for glycerol electrooxidation using the Pd_96_Fe_4_/GCN electrode with a mass activity of 1.86 A mg^−1^ metal, which is much better than that of the reported PdPt nanowires,^88^ carbon supported Pd based core–shell nanocatalysts,^91^ Pd_*x*_Bi catalysts,^93^ and metal oxide (CeO_2_, NiO, Co_3_O_4_ and Mn_3_O_4_)-supported Pd catalysts.^94^ Similarly, other Pd–Fe/GCN catalysts were tested for oxidation of methanol, ethylene glycol, tri-ethylene glycol, and glycerol and they followed a similar trend to ethanol oxidation, except for glycerol oxidation where the current density for Pd_85_Fe_15_/GCN was higher than that for Pd_91_Fe_9_/GCN (Table S3†). However, the current density further decreases significantly for the Pd_77_Fe_23_/GCN nanohybrids. In fact, Pd_96_Fe_4_/GCN showed high catalytic activity for oxidation of fuel such as methanol, ethylene glycol, tri-ethylene glycol, and glycerol.

Hence, Pd_96_Fe_4_/GCN showed the best catalytic activity among the different Pd–Fe compositions, which suggests that the introduction of Fe to form a binary system would allow gaining exceptionally high catalytic activity and stability. Among the Pd–Fe/GCN catalysts, such high catalytic activity of the Pd_96_Fe_4_/GCN catalyst may have originated from its porous structure, large surface area, and in particular the small size of each individual branch in the dendrites, which are required for a good catalyst.^38,39^ It may also be inferred that the substantially shorter metal–metal distances in the bimetallic nanoalloy catalysts and modification of the electronic structure of metals are responsible for their substantially improved catalytic properties, which is well consistent with the reported literature.^22^ Furthermore, the presence of Fe metal may contribute to the increase of resistance to poisoning of the Pd based catalytic surface with adsorption of chemical species as reported earlier.^20,95,96^ Additionally, the highly active sites and large effective surface area of carbon nanosheets with a graphene-like layered structure shorten paths for fast electrolyte ion diffusion, consequently offering more electron/charge transfer channels within the nanohybrid materials during catalysis.^97–99^ Furthermore, the combination of graphitic nanosheets with metal nanostructures leads to the protection of the metal nanocluster from further aggregation and provides superior catalytic activity, and can be extended as a scalable, clean approach without using any chemical reducing agents *via* radiolysis.

During ethanol oxidation, initially ethanol may adsorb on the electrode surface followed by oxidation and decomposition to generate intermediates such as acetate (CH_3_COO^−^), acetaldehyde (CH_3_CHO), and CO.^5^ In order to shed light on anodic oxidation of ethanol, a cyclic voltammetric study was carried out using Pd_96_Fe_4_/GCN electrodes immersed in 0.5 M KOH with sodium acetate, acetaldehyde and ethanol fuels (100 mM). Product analysis reveals that both acetaldehyde and acetate exist after electrooxidation of ethanol (Fig. S7, ESI†). Our preliminary results suggest that the oxide species adsorbed on the Pd_96_Fe_4_/GCN surface and Pd are converted into PdO due to CO being adsorbed, which could easily be desorbed to restore the active sites of Pd through transmetallation reaction between Pd and Fe. The regeneration of Pd active sites enhances ethanol oxidation and diminishes CO poisoning.

## 4. Conclusion

We have successfully demonstrated the simultaneous formation of graphitic nanosheets and deposition of homogeneously alloyed Pd and Fe bimetallic particles through radiolysis without using any additional stabilizing agents. The chemical composition and morphology of the Pd–Fe nanoalloy can be tuned by varying the initial concentration of Fe precursors. Transmission electron microscopy analysis reveals that the nanoparticles are strongly attached to the GCN surface, which makes the NP surface available for electrochemical reactions. Remarkably, the as-prepared Pd_96_Fe_4_/GCN catalyst exhibits extremely high catalytic activity for ethanol oxidation with high current density. The exceptionally high catalytic activity of Pd_96_Fe_4_/GCN could be mainly attributed to the strong synergistic effect between Pd and Fe atoms, and the large surface area of the 2D graphitic carbon nanosheet structures, which are enabled by enhancing the interactions between the graphene surface and metal species. The easy fabrication, cost-effectiveness, high intrinsic activity, and superior stability make the Pd–Fe/GCN nanohybrid a very promising catalyst for the electrochemical oxidation of alcohol. The present strategy would be useful to fabricate other graphitic carbon nanosheet supported multimetallic nanoalloys as superior anode catalysts for DAFC applications.

## Conflicts of interest

There are no conflicts to declare.

## Supplementary Material

NA-001-C9NA00317G-s001
