# Supplementary material for: Bimetallic Pd96Fe4 nanodendrites embedded in graphitic carbon nanosheets as highly efficient anode electrocatalysts
Source: Nanoscale Adv. 2019 Aug 19;1(10):3929–40. doi: 10.1039/c9na00317g (PMC9417808; doi:10.1039/c9na00317g)
Supplement: NA-001-C9NA00317G-s001 [file NA-001-C9NA00317G-s001.pdf]

Electronic Supplementary Material (ESI) for Nanoscale Advances.
This journal is © The Royal Society of Chemistry 2019

Electronic Supplementary Information

Bimetallic $Pd_{96}Fe_4$ Nanodendrites Embedded in Graphitic Carbon Nanosheets as Highly Efficient Anode Electrocatalysts

Srabanti Ghosh[a],* Sandip Bysakh[b], Rajendra Nath Basu[a]*

[a]*Fuel Cell and Battery Division, CSIR - Central Glass and Ceramic Research Institute,*
[b]*Materials Characterization Division, CSIR - Central Glass and Ceramic Research Institute*
196, Raja S. C. Mullick Road, Kolkata-700032, India

*Corresponding Authors Email: *ghosh.srabanti@gmail.com*

rnbasu@cgcri.res.in

List of contents:

Fig.S1 (a) FESEM image of mesocarbon microbeads (MCMB). (b) TEM and (c) HRTEM image of graphitic carbon nanosheets derived after radiolysis of MCMB solution.

Fig.S2 (a) TEM, (b) HRTEM, (c) HAADF-STEM images, and (d, e) elemental mapping and (f) EDS line scanning profiles of $Pd_{77}Fe_{23}$/GCN nanohybrids.

Fig.S3 A typical Raman spectrum of $Pd_{96}Fe_4$/GNC nanohybrid.

Fig.S4 Cyclic voltammetric runs associated with the electrocatalytic oxidation of 0.5 M EtOH by Fe_{100}/GCN in 1 M KOH. The reference electrode was Hg/HgO electrode. The scan rate was 50 mVs^{-1}.

Fig.S5 (a) Long cycling study of $Pd_{96}Fe_4$/GCN electrodes in a solution of 1M KOH and 0.5 M ethanol at scan rate of 50 mV Sec^{-1}.

Fig.S6 (a) Long cycling study of Pd/C, Pd/GCN, and $Pd_{96}Fe_4$/GCN electrodes in a solution of 1M KOH and 0.5 M ethanol at scan rate of 50 mV Sec^{-1}. (b) XRD, (c) FESEM (d) TEM images of $Pd_{96}Fe_4$/GCN electrodes after 1000 cycling of ethanol oxidation.

Fig.S7 Cyclic voltammograms for $Pd_{96}Fe_4$/GCN catalyst for CH_3CH_2OH, CH_3CHO, and CH_3COONa solutions fuels each of concentration 100 mM in 0.5 M aqueous KOH at a scan rate of 50 mV Sec^{-1}.

Table S1 Elemental compositions of Pd-Fe contained nanoalloys measured by ICP-AES.

Table S2 Comparison of the electrochemical performance of Pd electrocatalysts for the ethanol oxidation.

Table S3 Effect of catalysts for the oxidation of methanol, ethylene glycol, tri-ethylene glycol, glycerol.

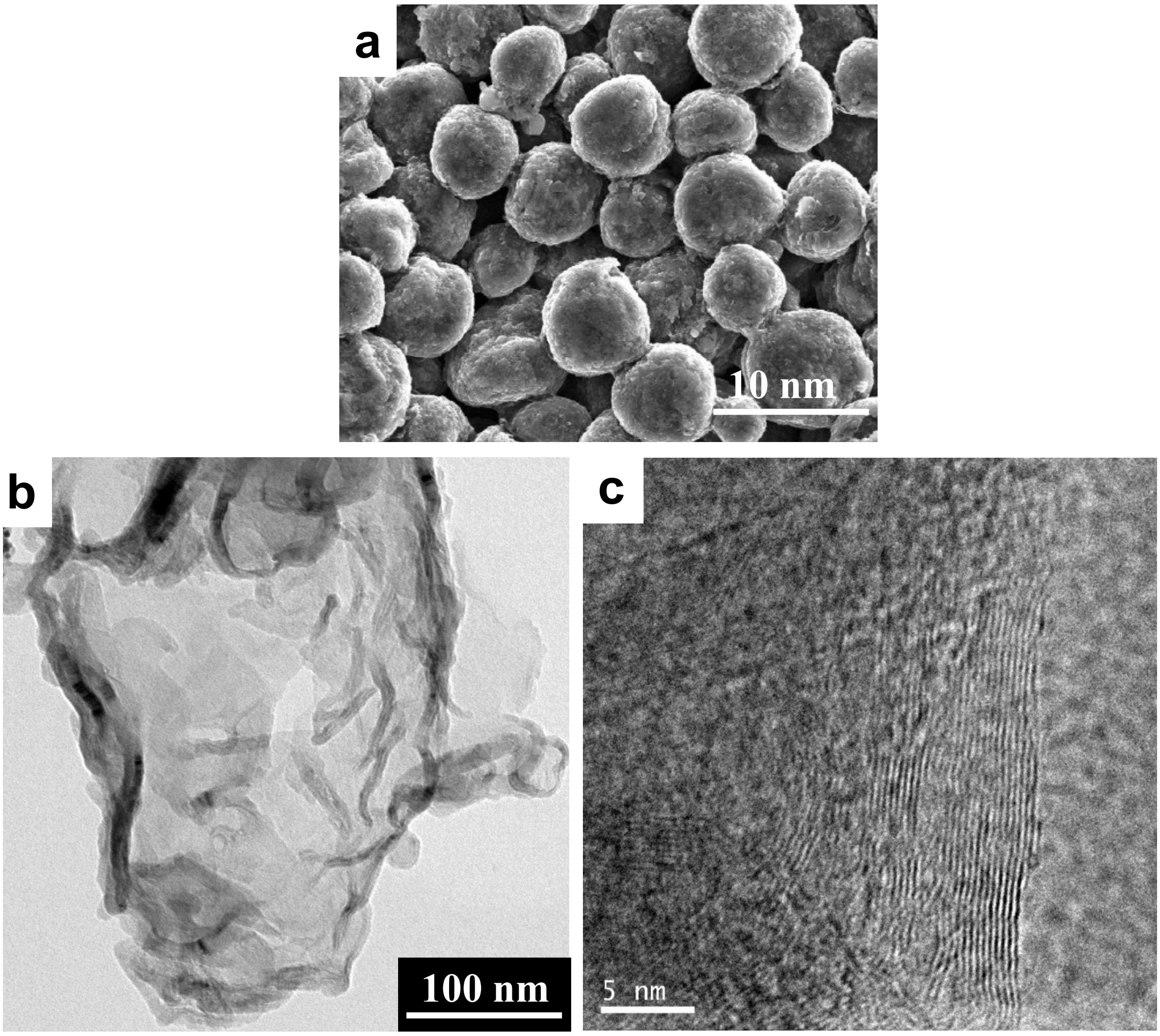

Fig.S1 (a) FESEM image of mesocarbon microbeads (MCMB). (b) TEM and (c) HRTEM image of graphitic carbon nanosheets derived after radiolysis of MCMB solution.

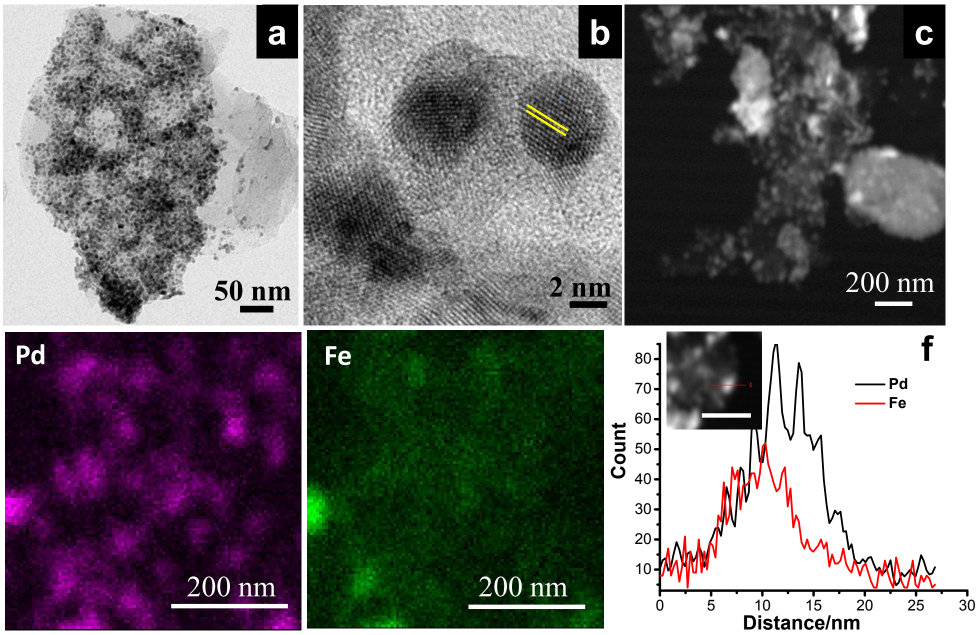

Fig.S2 (a) TEM, (b) HRTEM, (c) HAADF-STEM images, and (d, e) elemental mapping and (f) EDS line scanning profiles of $Pd_{77}Fe_{23}$/GCN nanohybrids.

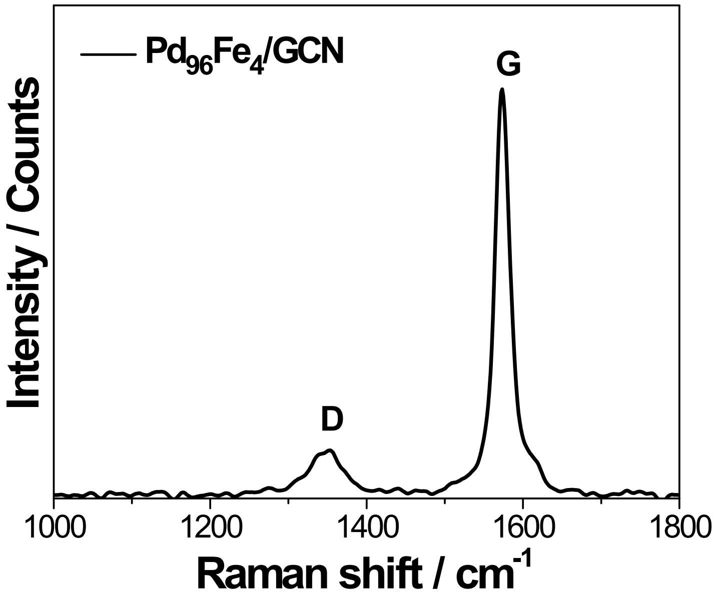

Fig.S3 A typical Raman spectrum of $Pd_{96}Fe_4$/GNC nanohybrid.

Table S1 Elemental compositions of Pd-Fe contained nanoalloys measured by ICP-AES.

Metal loaded on GCN	ICP-AES Atomic content (at.%)		Metal composition in solution (Atomic, at.%)		Metal content by ICP-AES Weight (%)
	Pd	Fe	Pd	Fe	
Pd	100	-	-	100	9±1%
Fe	-	100	100	-	1.5±0.35%
$Pd_{96}Fe_4$	96±2.1	4±1.5	90	10	4±0.15%
$Pd_{91}Fe_9$	91±3.2	9±1.8	85	15	3.4±.28%
$Pd_{85}Fe_{15}$	85±3.7	15±2.8	75	25	1.9±0.5%
$Pd_{77}Fe_{23}$	77±4.8	23±2.1	50	50	1.45±0.12%

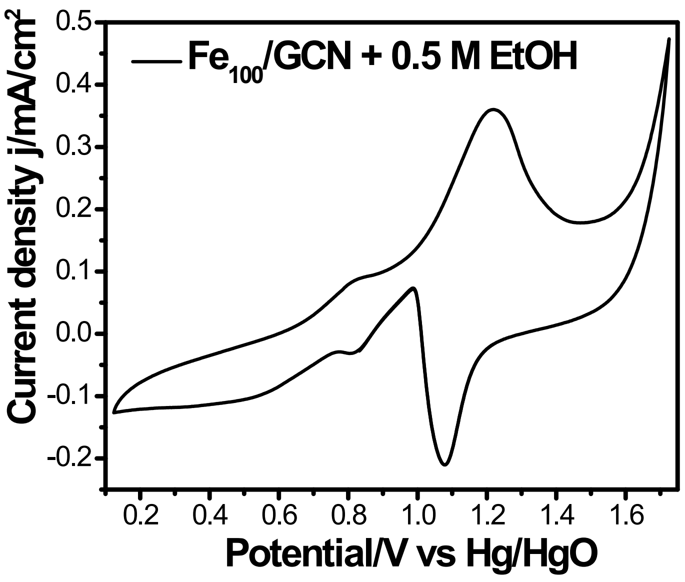

Fig.S4 Cyclic voltammetric runs associated with the electrocatalytic oxidation of 0.5 M EtOH by Fe_{100}/GCN in 1 M KOH. The reference electrode was Hg/HgO electrode. The scan rate was 50 mVs^{-1}.

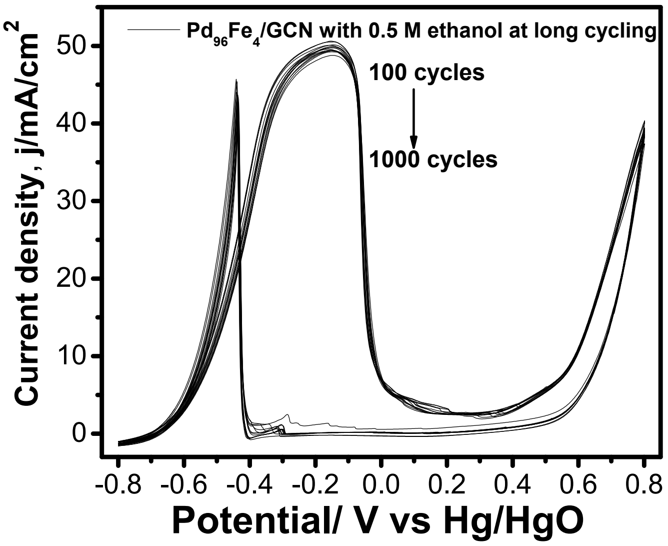

Fig.S5 (a) Long cycling study of $Pd_{96}Fe_4$/GCN electrodes in a solution of 1M KOH and 0.5 M ethanol at scan rate of 50 mV Sec^{-1}.

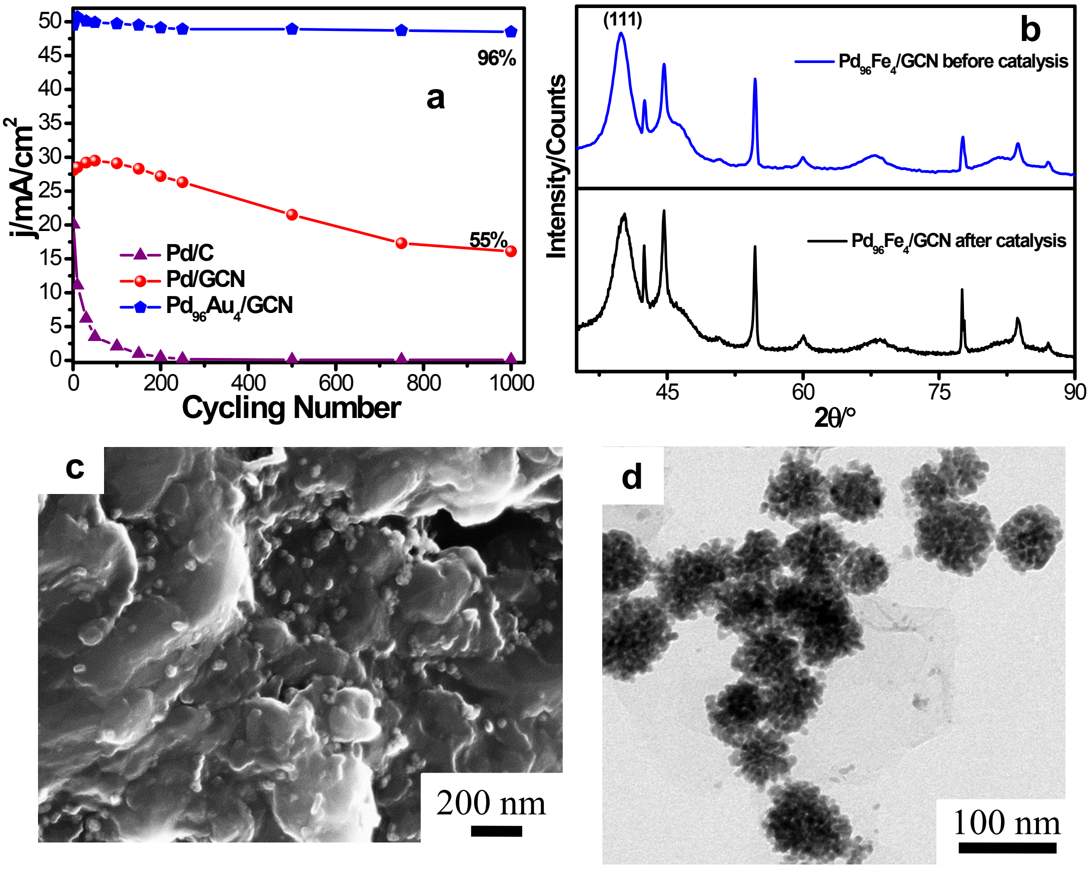

Fig.S6 (a) Long cycling study of Pd/C, Pd/GCN, and $Pd_{96}Fe_4$/GCN electrodes in a solution of 1M KOH and 0.5 M ethanol at scan rate of 50 mV Sec^{-1}. (b) XRD, (c) FESEM (d) TEM images of $Pd_{96}Fe_4$/GCN electrodes after 1000 cycling of ethanol oxidation.

Table S2 Comparison of the electrochemical performance of Pd electrocatalysts for the ethanol oxidation.

Electrode	E_{onset}, mV/RHE	j_f, $mA.cm^{-2}$	j_f, $mA.cm^{-2}.mg^{-1}$	Specific Current, j_f, $mA.mg^{-1}$	Reference
Pd black catalyst	–306	0.65	-	-	1
Pd/Graphene	–356	0.56	-	-	2
Pd/CNT	–320	-	364	-	3
Pd/C	–436	-	63	-	4
Pd/C	–335	-	42	-	5
Pd/Ppy	–384	7.05	4147	248.70	6
Pd/Graphene/Nafion	–376	14.22	5925	355.5	7
Pd/Nafion	–346	8.55	1745	104.7	7
Pd nanoplates/Nafion	–376	4.05	1500	90	8
Pd nanowires/Nafion	–420	-	1327	-	9
Pd/CNT	–426	-	3540	-	10
PtPdNPs/GNs	-	22.4	-	-	11
Ni@PbPt/Graphene	-	-	-	281	12
Pt−Pd (1:3)/RGO	-	-	-	1486.7	13
Pt–Cu/RGO	-	-	-	1114.7	14
PtPd NFs-RGO	-	-	-	600	15
PdCo NTAs/CFC	-	-	-	1491	16
Pd/PANI/Pd	-	-	-	310	17
Pd–PEDOT/GE	-	-	-	458.5	18
Pd–PEDOT	-	-	-	285.1	18
PtPd/PPy/PtPd nanotube	-	3.1	-	-	19
$Pd_{89}Pt_{11}$/PPy	–356	15.8	5197	782	20
$Pd_{54}Au_{46}$/PPy	–426	10.35	5280	792	20
$Pd_{96}Fe_4$/GCN	–486	49.8	11008	1100	This work

Table S3 Effect of catalysts for the oxidation of methanol, ethylene glycol, tri-ethylene glycol, glycerol.

**Fuel**	**Current density (mA/cm²)**				
	Pd_{100}/GCN	$Pd_{96}Fe_4$/GCN	$Pd_{91}Fe_9$/GCN	$Pd_{85}Fe_{15}$/GCN	$Pd_{77}Fe_{23}$/GCN
Ethanol	30.2	49.87	32.4	24.1	18.5
Methanol	1.87	5.38	4.7	4.4	4.023
Ethylene Glycol	12.8	22.63	7.9	7.8	3.48
Tri-Ethylene Glycol	0.88	1.29	0.84	0.33	0.13
Glycerol	2.75	8.37	7.08	7.70	4.3

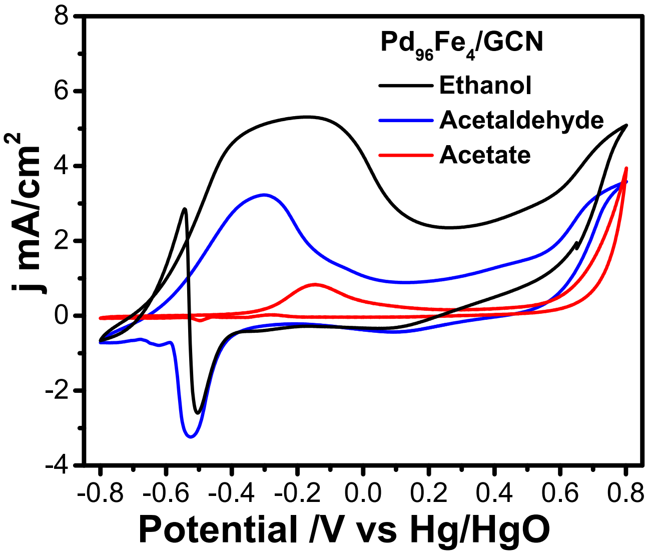

Fig.S7 Cyclic voltammograms for $Pd_{96}Fe_4$/GCN catalyst for CH_3CH_2OH, CH_3CHO, and CH_3COONa solutions fuels each of concentration 100 mM in 0.5 M aqueous KOH at a scan rate of 50 mV Sec^{-1}.

References

1. N. Tian, Z.-Y. Zhou, N.-F. Yu, L.-Y. Wang, S.-G. Sun, *J. Am. Chem. Soc.,* 2010, **132**, 7580–7581.
2. Q. Zhang, E. Uchaker, S. L. Candelaria and G. Cao, *Chem. Soc. Rev.,* 2013, **42**, 3127–3171.
3. N. Mackiewicz, G. Surendran, H. Remita, B. Keita, G. Zhang, L. Nadjo, A. Hagège, E. Doris and C. Mioskowski, *J. Am. Chem. Soc.,* 2008, **130**, 8110–8111.
4. C. Xu and P. K. Shen, Liu, Y. *J. Power Sources,* 2007, **164**, 527–531.
5. M. Grde´n, M. Łukaszewski, G. Jerkiewicz and A. Czerwi´nski, *Electrochim. Acta,* 2008, **53**, 7583–7598.
6. S. Ghosh, N. Bhandary, S. Basu and R. N. Basu, *Electrocatalysis*, 2017, **8**, 329–339.
7. S. Ghosh, H. Remita, P. Kar, S. Choudhury, S. Sardar, P. Beaunier, P. S. Roy, S. K. Bhattacharya and S. K. Pal, *J. Mater. Chem. A*, 2015, **3**, 9517–9527.
8. S. Ghosh, A.-L. Teillout, D. Floresyona, Pedro de Oliveira, A. Hagège and H. Remita, *Int. J. Hydrogen Energy*, 2015, **40**, 4951–4959.
9. F. Ksar, G. Surendran, L. Ramos, B. Keita, L. Nadjo, E. Prouzet, P. Beaunier, A. Hagège, F.; Audonnet and H. Remita, *Chem. Mater*. 2009, *21*, 1612–1617.
10. N. Mackiewicz, G. Surendran, H. Remita, B. Keita, G. Zhang, L. Nadjo, A. Hag`ege, E. Doris and C. Mioskowski, *J. Am. Chem. Soc*., 2008, **130**, 8110–8111.
11. X. Chen, Z. Cai, X. Chen and M. Oyama, *J. Mater. Chem. A,* 2014, **2**, 315–320.
12. D. Chen, Y. Zhao, Y. Fan, X. Peng, X. Wang and J. Tian, *J. Mater. Chem. A,* 2013, **1**, 13227–13232.
13. J.-J. Lv, N. Wisitruangsakul, J.-J. Feng, J. Luo, K.-M. Fang and A.-J. Wang, *Electrochim. Acta,* 2015, **160**, 100–107.
14. J.-J. Lv, J.-N. Zheng, L.-L. Chen, M. Lin, A.-J. Wang, J.-R. Chen and J.-J. Feng, *Electrochim. Acta,* 2014, **143**, 36–43.
15. M. Gong, Z. Yao, F. Lai, Y. Chen and Y. Tang, *Carbon*, 2015, **91**, 338–345.

16. A. L. Wang, X. J. He, X. F. Lu, H. Xu, Y. X. Tong and G. R. Li, *Angew. Chem. Int. Ed.,*2015, **54**, 3669–3673.

17. A.-L. Wang, H. Xu, J.-X. Feng, L.-X. Ding, Y.-X. Tong and G.-R. Li, *J. Am. Chem. Soc*., 2013, **135**, 10703−10709.

18. H. Xu, L.-X. Ding, C.-L. Liang, Y.-X. Tong and G.-R. Li, *NPG Asia Mater.,* 2013, **5**, e69–e72.

19. R. Yue, H. Wang, D. Bin, J. Xu, Y. Du, W. Lu and J. Guo, *J. Mater. Chem. A,* 2015, **3**, 1077–1088.

20. S. Ghosh, S. Bera, S. Bysakh and R. N. Basu, *ACS Appl. Mater. Interfaces*, 2017, **9**, 33775–33790.
